# Multi-societal expert consensus statement on the safe administration of ultrasound contrast agents

**DOI:** 10.1186/s44156-024-00068-7

**Published:** 2025-02-21

**Authors:** Jordan B. Strom, Andrew Appis, Richard G. Barr, Maria Cristina Chammas, Dirk-André Clevert, Kassa Darge, Linda Feinstein, Steven B. Feinstein, J. Brian Fowlkes, Beverly Gorman, Pintong Huang, Yuko Kono, Juan Lopez-Mattei, Andrej Lyshchik, Michael L. Main, Wilson Matthias, Christina Merrill, Sharon L. Mulvagh, Petros Nihoyannopoulos, Joan Olson, Fabio Piscaglia, Thomas Porter, Arnaldo Rabischoffsky, Roxy Senior, Jessica L. Stout, Maria Stanczak, Stephanie R. Wilson, the International Contrast Ultrasound Society with endorsement from the British Society of Echocardiography, the Canadian Society of Echocardiography, the Society of Diagnostic Medical Sonography, the Society for Pediatric Radiology, the World Federation of Ultrasound in Medicine and Biology, the Brazilian College of Radiology, the Joint Review Committee for Diagnostic Medical Sonography, the Chinese Ultrasound Doctors Association, the American Society of Neuroimaging and affirmation/support from the American Society of Echocardiography, the Association for Medical Ultrasound, and the Society for Vascular Ultrasound

**Affiliations:** 1 Richard A. and Susan F. Smith Center for Outcomes Research in Cardiology, Beth Israel Deaconess Medical Center, 375 Longwood Avenue, 4Th Floor, Boston, MA 02215 USA; 2 International Contrast Ultrasound Society, Chicago, USA; 3 Kaiser-Permanente, San Diego, USA; 4 Northeastern Ohio Medical University, Rootstown, USA; 5 University of São Paulo School of Medicine Clinics Hospital, São Paulo, Brazil; 6 Ludwig-Maximilians-Universität München, Munich, Germany; 7 Children’s Hospital of Philadelphia, Philadelphia, USA; 8 Department of Radiology, University of Michigan–Ann Arbor, Ann Arbor, USA; 9 Second Affiliated Hospital of Zhejiang University, Hangzhou, China; 10 University of California, San Diego, San Diego, USA; 11 Lee Heart Health Institute, Fort Myers, USA; 12 Thomas Jefferson University, Philadelphia, USA; 13 Saint Luke’s Mid America Heart Institute, Kansas City, USA; 14 University of Calgary, Calgary, Canada; 15 Dalhousie University, Halifax, Canada; 16 Imperial College London, London, UK; 17 University of Nebraska Medical Center, Omaha, USA; 18 Department of Medical and Surgical Sciences, University of Bologna, Bologna, USA; 19 Hospital Pró-Cardíaco, Rio de Janeiro, Brazil; 20 Royal Brompton Hospital, London, UK; 21 Division of Internal Medicine, Hepatobiliary and Immunoallergic Diseases, IRCCS Azienda Ospedaliero, Universitaria di Bologna, Italy, Bologna, Italy; 22 Dentons US, Chicago, USA

**Keywords:** Contrast ultrasound, Safety, Sonography, Guideline

## Abstract

Contrast enhanced ultrasound (CEUS) offers a safe, reliable imaging option to establish a clinical diagnosis across a variety of multidisciplinary settings. This Expert Consensus Statement serves to outline expert opinion on what constitutes appropriate supervision and the essential components of safe CEUS practice. The purpose of this document is to empower institutions to allow sonographers, along with other trained medical professionals, to administer UCAs at the point of care, consistent with the updated scope of practice documentation and within the broad parameters of an individual’s training and licensure, while subject to appropriate supervision and meeting or exceeding minimum safety standards. This guidance was developed by the International Contrast Ultrasound Society and endorsed by the following organizations that represent ultrasound professionals: the British Society of Echocardiography, the Canadian Society of Echocardiography, the Society of Diagnostic Medical Sonography, the Society for Pediatric Radiology, the World Federation of Ultrasound in Medicine and Biology, the Brazilian College of Radiology, the Joint Review Committee for Diagnostic Medical Sonography, the Chinese Ultrasound Doctors Association, and the American Society of Neuroimaging. Additionally, this guidance document was affirmed or supported by the American Society of Echocardiography, the Association for Medical Ultrasound, and the Society for Vascular Ultrasound.

## Preamble

Central to the provision of effective patient care is the ability to obtain a timely and accurate assessment of an individual’s clinical condition while maintaining the lowest risks for adverse outcomes. Contrast enhanced ultrasound (CEUS) offers a safe, reliable imaging option to establish a clinical diagnosis across a variety of multidisciplinary settings. CEUS studies use intracavitary or intravenously (IV)-injected ultrasound contrast agents (UCA, a.k.a. ultrasound enhancing agents), along with widely-available ultrasound hardware and software, to produce diagnostic images in real time [1–3]. The focus of this paper will be the CEUS via IV route. CEUS studies do not expose patients or staff to ionizing radiation, and UCAs are among the safest contrast agents used in modern medical imaging with no influence on renal or thyroid function [4]. CEUS studies can reduce the need for additional downstream testing [5–8], improve the time to diagnosis [9], lower overall imaging costs [5–7], and potentially improve workflows. There are also situations where CEUS may be the only suitable modality to evaluate an abnormality [10].

CEUS imaging has multiple applications in cardiology and radiology. UCAs are useful in diagnosing cardiac and vascular disease by improving endocardial border resolution and assessment of blood volume and myocardial perfusion [1, 2,9,11,12]. UCAs also enable identification and characterization of tumors, and monitoring of inflammatory and neoplastic gastro-intestinal and renal diseases. [1, 2, 9, 11, 12] In addition, novel applications of UCAs are being explored in the therapeutic realm, including molecular imaging, sonothrombolysis, and enhanced delivery of chemotherapy drugs and gene therapies [13, 14].

Although CEUS is underutilized relative to potential applications [5, 15], CEUS indications and use are growing in adult and pediatric patient populations worldwide. Reflecting that trend, numerous multi-societal guidance documents now support the administration of UCAs by sonographers and other medical professionals under appropriate supervision [1, 2, 9, 11, 12, 16]. For example, the recently updated “Scope of Practice and Clinical Standards for the Diagnostic Medical Sonographer,” promulgated by the Society of Diagnostic Medical Sonography (SDMS), allows a trained sonographer to determine when a CEUS examination is necessary, place an IV, and inject UCAs [17].

This Expert Consensus Statement serves to outline expert opinion on what constitutes appropriate supervision and the essential components of safe CEUS practice. The purpose of this document is to empower institutions to allow sonographers, along with other trained medical professionals, to administer UCAs at the point of care, consistent with the updated SDMS Scope of Practice [17] and within the broad parameters of an individual’s training and licensure, while subject to appropriate supervision and meeting or exceeding minimum safety standards. This document mirrors the new SDMS Scope of Practice [17] and is similar to practice parameters set forth by the American College of Radiology (ACR) [18] in predefining conditions for safe administration of iodinated contrast by non-physician personnel.

This guidance was developed by the International Contrast Ultrasound Society (ICUS) and endorsed by the following organizations that represent ultrasound professionals: the British Society of Echocardiography, the Canadian Society of Echocardiography, the Society of Diagnostic Medical Sonography, the Society for Pediatric Radiology, the World Federation of Ultrasound in Medicine and Biology, the Brazilian College of Radiology, the Joint Review Committee for Diagnostic Medical Sonography, the Chinese Ultrasound Doctors Association, and the American Society of Neuroimaging. Additionally, this guidance document was affirmed or supported by the American Society of Echocardiography, the Association for Medical Ultrasound, and the Society for Vascular Ultrasound. This document is intended solely as a general tool for educating and guiding medical professionals in the provision of safe, timely, and appropriate care within their scope of practice and institutional guidelines. This document is expressly not intended to establish a legal standard of care or to reflect appropriate clinical judgments or decisions made or to be made with respect to specific patients.

## Introduction

UCAs consist of liquid or lyophilized suspensions of microbubbles containing high-molecular weight gases surrounded by a phospholipid or albumin shell. Their small size (1.1–4.5 μm in diameter) permits unimpeded passage through the pulmonary and systemic microcirculation. When insonation is applied using a very low mechanical index (MI < 0.2), these microbubbles oscillate in a nonlinear fashion, emitting an ultrasound signal that can be used to create effective delineation of the blood pool and microvasculature.

UCAs are among the safest of all contrast media, and voluminous studies published over the past few decades support the overwhelming safety profiles of UCAs along with their clinical benefits for use in both cardiology and radiology applications [4, 19–30]. Nevertheless, on exceedingly rare occasions, serious immune-related reactions may occur, largely attributed to complement activation-related pseudoallergy (CARPA) reactions that result from interactions of the liposomal shell with the complement system, resulting in an anaphylactoid reaction that can be fatal if not addressed promptly [4, 19–30]. In multiple published large safety studies, CARPA reactions occur in approximately 1:15,000 UCA administrations and do not require prior exposure to the agent [4, 19–30]. In addition, rare IgE-mediated Type I hypersensitivity reactions, requiring prior exposure to an allergen, have been described to occur with components of the agent including the microbubble shell, the gaseous component, or excipients such as polyethylene glycol; use of these agents is contraindicated in individuals with a known allergy to any component [31]. Even more uncommonly, UCAs have been associated with Kounis Syndrome, the occurrence of an acute coronary syndrome in the setting of an allergic reaction [32–34], which is estimated to occur at a frequency of < 1/20,000 in the Food and Drug Administration Event Reporting System (FAERS) database [35]. While these rare reactions do not negate the multiple demonstrated clinical benefits of UCA use [9], they do highlight the need for guidance in safely administering these agents in clinical practice. It is important to note that UCAs have an improved safety profile compared with x-ray and MRI contrast agents [4].

### A. Adoption of protocol documents

We recommend that each facility adopt a written and standardized set of predefined imaging protocols for use of UCAs. These protocols should reflect consideration of the various UCAs available and indications or contraindications for each. These protocols should be predefined and the staff trained prior to the first use of UCAs at a given site. We recommend that these protocols stipulate:

1. Indications and contraindications for use.
2. The credentials required for administering healthcare professionals.
3. Procedures for administering and documenting use of UCAs including routinely documenting lot number and expiry date where feasible.
4. The institutional protocol for responding to an adverse reaction to contrast administration.
5. Procedures for response to adverse reactions.
6. The locations and contents of allergy kits (recommended to be available) and the procedure for use and restocking.
7. Which healthcare professionals may directly supervise administration of UCAs (see “E. Direct supervision of UCA administration”) and the appropriate hierarchy to seek medical assistance should this/these individual(s) not be available or reachable.
8. Procedures for documenting the occurrence of an adverse reaction, including retaining the vial for reporting of the lot number to the manufacturer, documenting the occurrence of an event in the study report and health record, counseling patients on future risks and avoidance of UCAs, and debriefing with staff around ways to improve future responses.

### B. Professional responsible for ordering CEUS study

The order for a CEUS imaging study should be placed by a licensed professional who meets institutional, state, and federal requirements for this task, potentially including physicians including residents or fellows or appropriately trained and licensed advanced practice professionals (nurse practitioner or physician assistant). The order should either be written or electronic, and should state the name of the ordering healthcare professional, the specific study ordered, and the date/time of the order. UCAs may be ordered as part of the overall order for a CEUS study or separately. When the option is available, the order for an ultrasound examination should include an order for UCAs to be injected at the discretion of the administering healthcare professional, so as to obviate the need for a separate order. A standing order within the ultrasound unit providing the appropriate indications for a CEUS study (see “A. Adoption of protocol documents”) allows administration of UCAs at the discretion of the trained healthcare professional performing the ultrasound examination after verbal consent from the patient.

### C. Professional responsible for performing the CEUS study

- The professional responsible for a CEUS study may or may not be different than the individual administering the UCA.
- Injections should follow societal guidelines as well as relevant institutional, state, and federal regulations around proper injection technique.
- Individuals who perform the CEUS study should be knowledgeable in cardiopulmonary resuscitation.
- Additionally, all individuals who perform the CEUS study should have access to an allergy kit and cardiopulmonary resuscitation cart (aka code cart).
- The professional should be familiar with the unit and institutional emergency/code procedures specifically including the immediate response to an adverse reaction to UCAs and the mechanism to activate an emergency response within their institution.
- Not superseding institutional, state, and federal regulations, injections of UCAs may be performed by licensed physicians and appropriately supervised medical personnel (see “D. Professional administering ultrasound contrast agent”).

### D. Professional administering ultrasound contrast agent

Not superseding institutional, state, and federal regulations, the following healthcare professionals may inject UCAs without (1) or with (2–5) direct supervision (see “E. Direct supervision of UCA administration”):

1. Physicians (MD, DO, MBBS)
2. Sonographers (RDMS, RDCS, RCS, ACS, RVT)
3. Advanced practice professionals (nurse practitioner or physician assistant)
4. Registered nurses
5. Other certified radiologic technologists or registered radiologist assistants

### E. Direct supervision of UCA administration

Under the general supervision of a licensed physician with training in UCA administration, the following healthcare professionals may also provide direct supervision of UCA administration:

1. Other physicians (MD/DO/MBBS) including residents and fellows
2. Advanced practice professionals (nurse practitioner or physician assistant)
3. Registered nurses
4. Sonographers acting in a supervisory capacity (i.e., lead sonographer, technical director, advanced sonographer)

The professional of direct supervision must be immediately available for assistance and direction throughout the procedure. Importantly, the direct supervisor is not required to be present in the room where and when the injection is performed. However, there should be at least one person meeting criteria for direct supervision in attendance (either in the room or in close proximity). This person should be knowledgeable of cardiopulmonary resuscitation techniques and institutional protocols for responding to adverse reactions and should be able to summon further medical support as needed. The professional directly supervising an injection should be competent in:

Recognition and immediate management of acute hypersensitivity reactions, including the types of reactions experienced in response to UCAs (see Introduction).

1. Local policies and protocols with regard to UCA administration and activation of the emergency response system (see “A. Adoption of protocol documents”).
2. Proficiency in cardiopulmonary resuscitation.

## Summary

This Expert Consensus Statement outlines guidelines around the safe administration of CEUS studies so that ultrasound units may confidently allow sonographers and other healthcare professionals to practice within their scope of practice, as permitted by institutional, state, and federal regulations, while ensuring the highest quality standard of patient care is met.

## Data Availability

No datasets were generated or analysed during the current study.

## References

[1] Mulvagh SL, Rakowski H, Vannan MA, et al. American society of echocardiography consensus statement on the clinical applications of ultrasonic contrast agents in echocardiography. J Am Soc Echocardiogr. 2008;21(11):1179–201. 10.1016/J.Echo.2008.09.009. 18992671 10.1016/j.echo.2008.09.009

[2] Senior R, Becher H, Monaghan M, et al. Clinical practice of contrast echocardiography: recommendation by the European association of cardiovascular imaging (Eacvi) 2017. Eur Heart J Cardiovasc Imaging. 2017;18(11):1205–1205af. 10.1093/Ehjci/Jex182. 28950366 10.1093/ehjci/jex182

[3] Hampson R, Senior R, Ring L, et al. Contrast echocardiography: a practical guideline from the british society of echocardiography. Echo Res Pract. 2023;10(1):23. 10.1186/S44156-023-00034-9. 37964335 10.1186/s44156-023-00034-9 PMC10648732

[4] Kaul S, Wei K. When you have eliminated the impossible, whatever remains, however improbable, must be the truth. Eur J Echocardiogr. 2009;10(6):713–5. 10.1093/Ejechocard/Jep102. 19671627 10.1093/ejechocard/jep102

[5] Strom Jordan B, Song Y, Jiang W, et al. Predictors and utilization of ultrasound enhancing agents for echocardiography in the outpatient setting. Jacc. 2024. 10.1016/J.Jcmg.2023.08.007. 37930284 10.1016/j.jcmg.2023.08.007

[6] Kurt M, Shaikh KA, Peterson L, et al. Impact Of contrast echocardiography on evaluation of ventricular function and clinical management in a large prospective cohort. J Am Coll Cardiol. 2009;53(9):802–10. 10.1016/J.Jacc.2009.01.005. 19245974 10.1016/j.jacc.2009.01.005

[7] Wei K, Peters D, Belcik T, et al. A predictive instrument using contrast echocardiography in patients presenting to the emergency department with chest pain and without St-segment elevation. J Am Soc Echocardiogr. 2010;23(6):636–42. 10.1016/J.Echo.2010.03.013. 20418056 10.1016/j.echo.2010.03.013 PMC2876194

[8] Lee KC, Liu S, Callahan P, et al. Routine use of contrast on admission transthoracic echocardiography for heart failure reduces the rate of repeat echocardiography during index admission. J Am Soc Echocardiogr. 2021;34(12):1253-1261.E4. 10.1016/J.Echo.2021.07.008. 34284098 10.1016/j.echo.2021.07.008

[9] Am F, Jb S. Impact Of ultrasound enhancing agents on clinical management. Curr Opin Cardiol. 2022;37(5):389–93. 10.1097/Hco.0000000000000973. 35913366 10.1097/HCO.0000000000000973 PMC9378600

[10] Schwarze V, Marschner C, Figueiredo G, Rübenthaler J, Clevert D-A. 2019; Single-Center Study: Evaluating The Diagnostic Performance And Safety Of Contrast-Enhanced Ultrasound (Ceus) In Pregnant Women To Assess Hepatic Lesions. *Ultraschall In Der Medizin*. 10.1055/A-0973-8517 10.1055/a-0973-8517 31362328

[11] Tr P, Sl M, Ss A, et al. Clinical applications of ultrasonic enhancing agents in echocardiography: 2018 American society of echocardiography guidelines update. J Am Soc Echocardiogr. 2018;31(3):241–74. 10.1016/J.Echo.2017.11.013. 29502588 10.1016/j.echo.2017.11.013

[12] Porter TR, Feinstein SB, Senior R, et al. Ceus cardiac exam protocols international contrast ultrasound society (Icus) recommendations. Echo Res Pract. 2022;9(1):7. 10.1186/S44156-022-00008-3. 35996167 10.1186/s44156-022-00008-3 PMC9396906

[13] Porter Thomas R, Smith Lynette M, Wu J, et al. Patient outcome following 2 different stress imaging approaches. J Am Coll Cardiol. 2013;61(24):2446–55. 10.1016/J.Jacc.2013.04.019. 23643501 10.1016/j.jacc.2013.04.019

[14] Pa D, Ljm J, Rjp M, et al. Microbubbles and ultrasound: from diagnosis to therapy. Eur J Echocardiogr. 2004;5(4):245–6. 10.1016/J.Euje.2004.02.001. 15219539 10.1016/j.euje.2004.02.001

[15] Fraiche AM, Manning WJ, Nagueh SF, Main ML, Markson LJ, Strom JB. Identification of need for ultrasound enhancing agent study (the in-use study). J Am Soc Echocardiogr. 2020. 10.1016/J.Echo.2020.07.015. 32919859 10.1016/j.echo.2020.07.015 PMC7722181

[16] Mulvagh SL, Mitchell C, Bagley J, et al. A survey of ceus education in sonographer training programs: the well-opacified and delineated road less travelled, and ready to be taken. J Am Soc Echocardiogr. 2020;33(9):A19–21. 10.1016/J.Echo.2020.07.010. 32891259 10.1016/j.echo.2020.07.010

[17] Scope Of Practice And Clinical Standards For The Diagnostic Medical Sonographer. *J Diagn Med Sonogr*. 2024; 40(3):312–328. 10.1177/87564793241242633

[18] Radiology Aco. Acr-Spr practice parameter for the use of intravascular contrast media. 2024. https://www.acr.org/-/Media/Acr/Files/Practice-Parameters/Ivcm.Pdf. Accessed 23 April 2024.

[19] Main ML, Ryan AC, Davis TE, Albano MP, Kusnetzky LL, Hibberd M. Acute mortality in hospitalized patients undergoing echocardiography with and without an ultrasound contrast agent (multicenter registry results in 4,300,966 consecutive patients). Am J Cardiol. 2008;102(12):1742–6. 10.1016/J.Amjcard.2008.08.019. 19064035 10.1016/j.amjcard.2008.08.019

[20] Exuzides A, Main ML, Colby C, Grayburn PA, Feinstein SB, Goldman JH. A retrospective comparison of mortality in critically ill hospitalized patients undergoing echocardiography with and without an ultrasound contrast agent. Jacc Cardiovasc Imaging. 2010;3(6):578–85. 10.1016/J.Jcmg.2010.04.006. 20541713 10.1016/j.jcmg.2010.04.006

[21] Kusnetzky LL, Khalid A, Khumri T, Moe TG, Jones PG, Main ML. Acute mortality in hospitalized patients undergoing echocardiography with and without an ultrasound contrast agent: results in 18,671 consecutive studies. J Am Coll Cardiol. 2008;51(17):1704–6. 10.1016/J.Jacc.2008.03.006. 18436124 10.1016/j.jacc.2008.03.006

[22] Abdelmoneim Sahar S, Bernier M, Scott Christopher G, et al. Safety of contrast agent use during stress echocardiography. Cardiovasc Imaging. 2009;2(9):1048–56. 10.1016/J.Jcmg.2009.03.020. 10.1016/j.jcmg.2009.03.020 19761981

[23] Gabriel RS, Smyth YM, Menon V, et al. Safety of ultrasound contrast agents in stress echocardiography. Am J Cardiol. 2008;102(9):1269–72. 10.1016/J.Amjcard.2008.06.066. 18940305 10.1016/j.amjcard.2008.06.066

[24] Shaikh K, Chang S, Peterson L, et al. Safety of contrast administration for endocardial enhancement during stress echocardiography compared with noncontrast stress. Am J Cardiol. 2008;102(11):1444–50. 10.1016/J.Amjcard.2008.07.032. 19026293 10.1016/j.amjcard.2008.07.032

[25] Oa K, Ka S, Al-Mallah Mh. Meta-analysis of adverse cardiovascular events associated with echocardiographic contrast agents. Am J Cardiol. 2010;106(5):742–7. 10.1016/J.Amjcard.2010.04.034. 20723656 10.1016/j.amjcard.2010.04.034

[26] Tang C, Fang K, Guo Y, et al. Safety of sulfur hexafluoride microbubbles in sonography of abdominal and superficial organs: retrospective analysis of 30,222 cases. J Ultrasound Med. 2017;36(3):531–8. 10.7863/Ultra.15.11075. 28072475 10.7863/ultra.15.11075

[27] Piscaglia F, Bolondi L. The safety of sonovue in abdominal applications: retrospective analysis of 23188 investigations. Ultrasound Med Biol. 2006;32(9):1369–75. 10.1016/J.Ultrasmedbio.2006.05.031. 16965977 10.1016/j.ultrasmedbio.2006.05.031

[28] Piskunowicz M, Kosiak W, Batko T, Piankowski A, Połczyńska K, Adamkiewicz-Drożyńska E. Safety Of intravenous application of second-generation ultrasound contrast agent in children: prospective analysis. Ultrasound Med Biol. 2015;41(4):1095–9. 10.1016/J.Ultrasmedbio.2014.11.003. 25701526 10.1016/j.ultrasmedbio.2014.11.003

[29] Darge K, Papadopoulou F, Ntoulia A, et al. Safety Of contrast-enhanced ultrasound in children for non-cardiac applications: a review by the society for pediatric radiology (Spr) and the international contrast ultrasound society (Icus). Pediatr Radiol. 2013;43(9):1063–73. 10.1007/S00247-013-2746-6. 23843130 10.1007/s00247-013-2746-6

[30] Papadopoulou F, Ntoulia A, Siomou E, Darge K. Contrast-enhanced voiding urosonography with intravesical administration of a second-generation ultrasound contrast agent for diagnosis of vesicoureteral reflux: prospective evaluation of contrast safety in 1,010 children. Pediatr Radiol. 2014;44(6):719–28. 10.1007/S00247-013-2832-9. 24442338 10.1007/s00247-013-2832-9

[31] Lindner A Jr, Belcik T, Main M, et al. Expert consensus statement from the american society of echocardiography on hypersensitivity reactions to ultrasound enhancing agents in patients with allergy to polyethylene glycol. J Am Soc Echocardiogr. 2021;34(7):707–8. 10.1016/J.Echo.2021.05.002. 33971277 10.1016/j.echo.2021.05.002 PMC8263497

[32] Yopes MC, Larnard EA, Liu SD, et al. Kounis syndrome after administration of ultrasound enhancing agent. Circulation. 2024;17(3):E016362. 10.1161/Circimaging.123.016362. 38415350 10.1161/CIRCIMAGING.123.016362 PMC10950526

[33] Sagalov A, Eggert A, Rimawi A, Hegde S. A rare presentation Of Kounis syndrome induced By an echocardiography contrast. Cjc Open. 2023;5(10):757–9. 10.1016/J.Cjco.2023.07.009. 37876888 10.1016/j.cjco.2023.07.009 PMC10591133

[34] Portero-Portaz JJ, Córdoba-Soriano JG, Gallego-Page JC. Type Iii Kounis syndrome after administration of an echocardiography contrast agent. Eur Heart J Acute Cardiovasc Care. 2020. 10.1177/2048872616655943. 27325844 10.1177/2048872616655943

[35] Administration Fad. Fda Adverse Event Reporting System (Faers) Public Dashboard. Accessed April 23, 2024, 2024. https://Www.Fda.Gov/Drugs/Questions-And-Answers-Fdas-Adverse-Event-Reporting-System-Faers/Fda-Adverse-Event-Reporting-System-Faers-Public-Dashboard

